# The Development and Evaluation of Insect Traps for the Asian Citrus Psyllid, *Diaphorina citri* (Hemiptera: Psyllidae), Vector of Citrus Huanglongbing

**DOI:** 10.3390/insects13030295

**Published:** 2022-03-16

**Authors:** James Snyder, Katrina L. Dickens, Susan E. Halbert, Stefanie Dowling, Dyrana Russell, Ruth Henderson, Eric Rohrig, Chandrika Ramadugu

**Affiliations:** 1 Florida Department of Agriculture and Consumer Services, Division of Plant Industry, Gainesville, FL 32608, USA; james.snyder@fdacs.gov (J.S.); katrina.dickens@fdacs.gov (K.L.D.); susan.halbert@fdacs.gov (S.E.H.); dowling_graphics@yahoo.com (S.D.); dyrana.russell@fdacs.gov (D.R.); eric.rohrig@fdacs.gov (E.R.); 2 USDA APHIS PPQ S&T, Salinas, CA 93905, USA; ruth.henderson@usda.gov; 3 Department of Botany and Plant Sciences, University of California Riverside, Riverside, CA 92521, USA

**Keywords:** psyllid trap, 3D printing, ACP, vector monitoring, huanglongbing, greening, citrus

## Abstract

**Simple Summary:**

Citrus cultivation is affected in many parts of the world because of a devastating disease, huanglongbing (HLB) or citrus greening. In Florida, nearly all commercial citrus is compromised due to HLB, and the disease has spread to other citrus-growing regions of the United States, California and Texas. In California, testing Asian citrus psyllids (ACPs) for the HLB pathogen has been an essential part of integrated pest management. ACP and HLB surveys are essential for disease management in areas where HLB is not widespread. We developed improved ACP traps that can be deployed in the field along with the standard yellow sticky traps. The reusable traps were designed with Rhinoceros computer software and a 3D printer. These traps can be deployed for several months and provide a dynamic sampling mechanism for an improved disease survey strategy. In the present study, ACPs from the 3D-printed traps are collected in a preservative and appear suitable for HLB testing. The evaluation of traps in Florida and California under laboratory, greenhouse, and field conditions indicates that the 3D-printed traps can capture ACPs with about the same efficiency as the sticky traps. They are easy to handle and provide an important field tool for HLB management.

**Abstract:**

Citrus huanglongbing (HLB) is a severe problem for citrus cultivation. The disease management programs benefit from improved field tools suitable for surveying the ACP vector (Asian citrus psyllid, *Diaphorina citri* Kuwayama (Hemiptera: Psyllidae)) and the associated pathogen. In the present study, we utilize three-dimensional (3D) printers and design tools to develop traps that can capture and preserve ACPs. Three novel, 3D-printed traps were designed and evaluated: stem trap, and cylinder traps 1 and 2. The traps and yellow sticky cards were deployed weekly for 8 months in 2 non-commercial citrus groves in Florida; in California, the traps were evaluated for 12 months in field cages and 4 citrus groves. The stem traps captured lower numbers of ACPs at all experimental sites compared to the cylinder traps. Capture rates in the cylinder traps were comparable to the sticky trap, making the device a viable tool for monitoring field ACPs. The two main advantages of using the reusable 3D traps over standard methods of ACP and HLB surveys include dynamic sampling that can be conducted year-round and the capture of ACPs that can be preserved and tested. Improved trapping may facilitate quick management decisions and mitigate HLB.

## 1. Introduction

Huanglongbing (HLB) or citrus greening is considered the most serious disease of citrus worldwide, because of the extensive damage to citrus industries. The challenges are associated with the difficulties in early diagnosis, the rapid spread of the disease by prolific insect vectors that are difficult to control, the lack of an effective cure, and the financial costs of managing the disease [1]. Citrus HLB was known in Asia for over a hundred years and was detected in the western hemisphere in 2004 [2,3]. In the United States, the first report of HLB was in 2005 [4]. Citrus production in Florida decreased by 74% since the disease was introduced in 2005 [5]. A concomitant reduction in the number of citrus groves, juice processing facilities, and packing houses indicates a dire situation for the Florida citrus industry [6]. In Florida, substantial economic losses correlated with HLB have been reported [7,8]. The disease is now present in Texas and California, other prominent citrus growing regions of the United States [9,10]. The development of strategies that improve disease management is essential for coping with the economic challenges of HLB.

The Asian citrus psyllid (ACP), *Diaphorina citri* Kuwayama (Hemiptera: Psyllidae) [11], is a phytophagous insect that feeds primarily on the phloem of host plants and is a vector of the bacterium *Candidatus* Liberibacter asiaticus (*C*Las), associated with HLB [2,12]. This unculturable bacterium invades the phloem of the host plant and contributes to an overall decline of plant health [13]. The control of ACP populations has been central to disease mitigation, especially in regions where the insect vector proliferates, but the disease has not been established. Vector control may not reduce disease prevalence in places, such as Florida, where 100% HLB incidence is estimated; however, it may be effective for controlling HLB in regions where the disease is not widespread [14]. In California, HLB has not yet established in commercial citrus, but psyllids are found in several locations. A state-wide coordinated response to control vector populations is recommended by the California Citrus Pest and Disease Prevention Committee (CPDPC) [15]. Since HLB disease control is currently dependent on vector control, the development of tools for efficient ACP surveys are useful.

The CLas pathogen has been reported from the insect vectors present in a citrus grove six years before disease was detected in the plant hosts [16,17]. Since a single bacteriferous adult ACP can inoculate the pathogen into the host plant and start the disease cycle [18], it is essential to monitor the ACP for the HLB pathogen. The early detection of *C*Las in ACP provided timely warnings about disease spread in citrus groves in various geographical regions, and in nurseries, long before symptom development [16,19,20]. Yellow sticky traps are useful for monitoring ACP populations, but the captured ACPs are degraded on the sticky surface, making them unsuitable for reliable pathogen testing [21]. In addition, adhesive traps have a high level of non-target by-catch, are very messy, difficult to handle, and are inconvenient to use in the field and the laboratory [22,23]. Due to degradation of ACPs captured on sticky cards, the DNA extracted is of lower quality compared to other preservation methods [24]. Alternative trapping systems that are easy to deploy, reduce non-target by-catch, do not generate the sticky mess, and preserve specimens for molecular analysis are valuable [24].

Another commonly used method is the manual capture of ACPs using aspirators, storage in alcohol, and laboratory testing for the pathogen [25]. This method is laborious, and it is extremely challenging to conduct a high throughput screening of ACP vectors for the presence of *C*Las. To develop tools essential for large-scale HLB surveys and capture ACP that can be preserved, we designed plastic traps printed with a 3D printer. The Florida Department of Agriculture and Consumer Services used desktop 3D printers to develop a target-specific trap that is highly attractive to ACPs. The 3D printers print objects designed on computer software using melted plastic extruded in successive layers to create the object. Traps can be designed and printed in-house, evaluated under laboratory conditions and modified as needed through an iterative process. The final design can also be printed using commercial 3D-printing services.

In the present study, we describe the development of ACP traps for efficient capture in field conditions. The 3D-printed traps provide a method to capture insects that can be tested for the presence of Liberibacter pathogens [24,26]. The traps were evaluated in the field conditions in Florida. In California, the traps were tested in field cages and citrus groves. The performance of the three trap models was compared with each other and with the standard yellow sticky traps. The 3D-printed traps provide several advantages, including dynamic ACP surveys and the capture of well-preserved ACPs. The information generated may be useful for better management of this economically significant disease.

## 2. Materials and Methods

### 2.1. ACP Traps

We designed insect traps, printed them using a 3D printer, and evaluated them for ACP capture in laboratory and field conditions. The two basic designs described in this work are “stem” traps and “cylinder” traps. All traps included a main body with a yellow-colored exterior to attract ACPs visually. The traps have entry holes either on top of the main body or at different levels on the unit’s exterior surface to facilitate the entry of ACPs inside the trap. The interior surface of the trap is slippery and connected at the bottom to a capture vial filled with a preservative. All traps were fitted with a clear plastic dome, about 100 mm in diameter, to allow sunlight to pass through. The light inside the traps attracts ACPs into the dome and then into the trap’s body. The interior surfaces were made slippery by applying a milky white fluoropolymer resin suspension of polytetrafluoroethylene (“Fluon”, marketed as “Insect-a-Slip”; BioQuip, Inc., Rancho Dominguez, CA, USA). It is essential to have sunlight penetrate the trap through the dome since ACP is attracted to light. We reapplied “Fluon^®^” as needed to maintain the slippery texture on the interior surface of the trap. A plastic cover was fitted at the bottom of the cylindrical traps to exclude spiders and other arthropods near the base of the trap. After each experiment, or at least once in eight weeks, the traps were washed with detergent and water to clean the body of the trap, and remove dirt and grime from the clear dome.

### 2.2. Stem Traps

**Figure 1:**
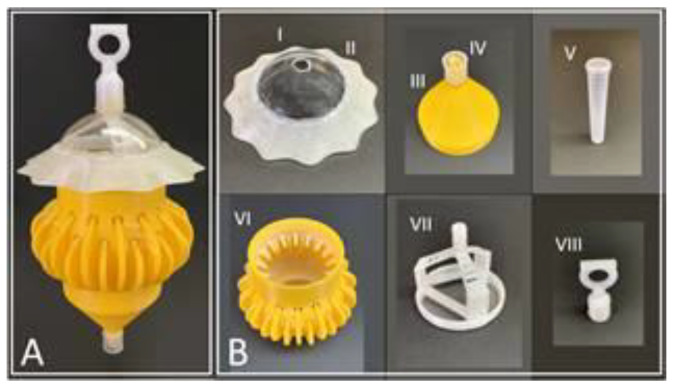
Stem trap. (**A**) Assembled stem trap used for the study. (**B**) Parts of the stem trap: I. Semi-circular, clear, plastic dome with an aperture at the top; II. Rain skirt; III. Funnel structure; IV. Funnel outlet for connecting the cap of the capture vial; V. Capture vial; VI. Body of the trap with stem-like protrusions on the outer surface leads to entry holes to guide ACPs into the trap’s interior; VII. Tripod structure; and VIII. Hanging nut used to deploy the trap and connect the tripod structure with the dome.

The stem trap (Figure 1) was designed based on our observations that ACP walks along stem-like structures. The traps had 2 rows of 16 equidistant circular holes (11 mm) intersected by 4 mm stem-like projections, which extended from the bottom exterior surface of the trap (Figure 1A). The projections passed through the center of each entry hole and were connected to the interior surface of the trap. ACPs land on the exterior surface of traps, walk up these stems leading them into the body of the trap. A tripod hanger screwed into the top of the trap body was fitted with a plastic bolt at the center. A rain skirt was printed using a 3D printer and hot-glued onto the dome to prevent rainwater from entering the trap’s interior through the entry holes. A 10 mm hole was drilled on top of the clear plastic dome, and a hanging nut was hot-glued on top. The dome was screwed on the top, and a funnel was attached to the bottom of the trap body. The preservation vial caps (Thermo Fisher Scientific, Inc., 2019, Cat. No. 14-956-7E, Pittsburg, PA, USA) were drilled and hot-glued onto the bottom of the trap funnel. Just before the trap deployment, capture vials containing preservation fluid were fitted into the caps. All the components were printed using a 3D printer in the stem traps, except the clear plastic dome at the top and the capture tubes attached to the funnel. A previous version of the stem traps described here was used for the trapping of the tomato psyllid, *Bactericera cockerelli* [22,24]; the stem trap described in the current manuscript has many novel features and modifications compared to the one described earlier in the tomato psyllid study.

### 2.3. Cylinder Traps

**Figure 2:**
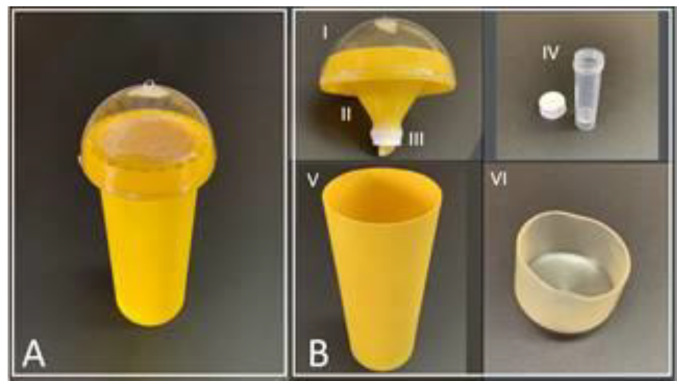
Cylinder trap 1. (**A**) Fully assembled unit. (**B**) Parts for the cylinder trap: I. Semi-circular plastic dome with a wire loop at the apical part for deploying the trap; II. Funnel with entry holes near the rim; III. The outlet of the funnel for connecting the cap of the capture vial; IV. Capture vial and cap; V. Body of the trap is sanded and painted bright yellow; and VI. Rubber covering for the open bottom end of the cylindrical body of the trap.

Cylinder traps were assembled using commercially available parts in combination with custom-made funnels printed using a 3D printer (Figure 2). The external surface of the traps consisted of clear, cylindrical, plastic postal tubes (three-inch diameter, obtained from Uline Inc., Catalog #S-14121, Pleasant Prairie, WI, USA). These were cut into eight-inch-long pieces and sanded (3M Center, 100 grit: 332U, 320 grit: 738U, St. Paul, MN, USA) to facilitate better paint adhesion. Sanded tubes were treated with yellow spray paint (Rust-Oleum, 7747, sunburst yellow, Vernon Hills, IL, USA), fluorescent paint containing barium sulfate (Krylon, 3104, fluorescent lemon-yellow, Cleveland, OH, USA), and a liquid rubberized coating (Clear Flex Seal, Swift Inc., 2019, Weston, FL, USA). This created a treated surface on which ACP could easily move (monitored in a laboratory setting). The design features of the 3D printed funnel include (1) entry holes, (2) overhang, and (3) funnel. Twenty-four equidistant entry holes (4 mm × 4 mm) allowed adult ACPs to enter the trap funnel. Due to the size of the orifice, larger insects that could not squeeze through the holes were eliminated from the capture tubes. The rim of the trap extruded downward 20 mm, creating a 4 mm thick overhang that provided shade for the entry holes and reduced the amount of light that entered the trap from below. The 3D-printed funnel was designed to pressure fit into the painted cylinder, and a clear plastic dome was hot-glued on top. A small hole was drilled into the top of the transparent dome, and a small ring of metal wire was hot-glued on the top to facilitate the deployment of the trap on a citrus tree.

Plastic self-standing transport tubes with screw caps were utilized to collect and preserve captured insects (Thermo Fisher Scientific, Cat. No. 22-010-1228, Pittsburg, PA, USA). A clear rubber cap (Uline, S-15014, Pleasant Prairie, WI, USA) was pressed to the underside of each cylinder trap to keep the collection vial area clean and free from other arthropods. The vial cap was drilled and hot-glued onto the bottom of the trap funnel.

Adult ACPs are attracted to the yellow exterior, land on the trap’s surface, and move upward towards the entry holes that appear lit from the outer surface of the trap. Once ACPs enter the trap, the viewing angle changes, and because of the added shading mechanism mentioned before, if the ACPs were to look back towards the entry holes, they would appear darkened and hence unappealing.

**Figure 3:**
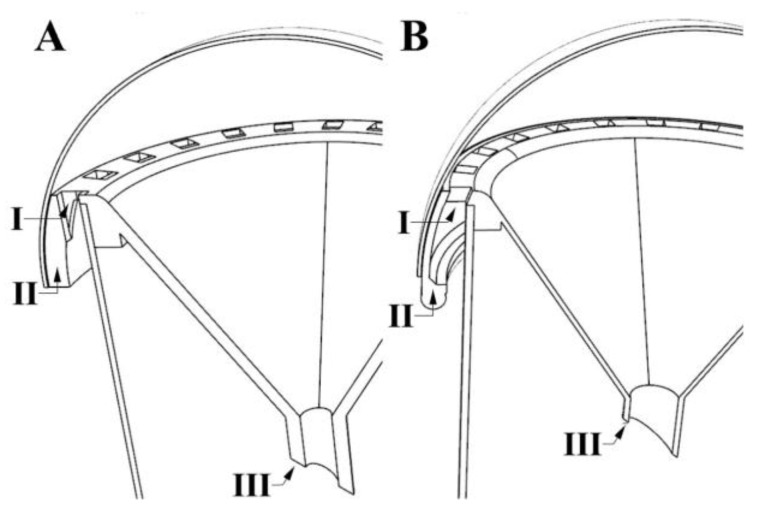
Diagram showing the differences between cylinder traps 1 (panel (**A**)) and 2 (panel (**B**)). I. Funnel rim with 24 equidistant entry holes; in trap 2, they are at a 35–45° angle. II. The funnel rim has an extra ledge in trap 2. III. The thickness of the plastic material used to print the funnel is 3 mm in trap 1 and 1.5 in trap 2. The opening of the funnel leads to a capture tube.

Two versions of the cylinder trap were evaluated in this study. The original cylinder trap 1 was modified into cylinder trap 2 by incorporating three significant changes: (1) entry holes were angled inward at 35–45°, (2) the bottom interior surface of the external brim structure was curved up and inward to create a sill, and (3) the thickness of the 3D-printed funnel was reduced from 3 mm to 1.5 mm. Reduced filament was required to print the funnel in cylinder trap 2 (Figure 3). The sill designed at the bottom of the brim helps land ACPs that may find their way out from the trap. Landing allows ACPs to reorient and climb back up into the trap. In the cylinder traps, only the funnel part with the entry holes was printed using a 3D printer; the rest of the parts of the trap were purchased from commercial sources.

### 2.4. 3D Design and Printing

Traps were designed using Rhinoceros, a computer-aided design software to produce a stereolithography (STL) file (Robert McNeel & Associates 2019, Seattle, WA, USA). This was converted to a G-code using Cura LulzBot Edition slicing software (Aleph Objects, Inc., 2019, Loveland, CO, USA) and printed using LulzBot Taz 5 and 6 fused depositional desktop 3D printers (Aleph Objects, Inc., 2019, Loveland, CO, USA). Polylactic Acid (PLA) plastic filament (Polymaker LLC, 2018, PolyLite™, Bluffton, SC, USA) in a “True Yellow” shade was selected for printing as it most closely matched the yellow-colored traps successfully used in other ACP trapping studies [27,28].

### 2.5. Field Trials in Florida

Field experiments in Florida were conducted in two non-commercial citrus groves: Ocklawaha (29.037281° N, 29.037013° W) and Merritt Island (28.464417° N, 80.712989° W). We used a randomized complete block design and designated eight blocks for trap assessments at each of the two sites. The four treatments consisted of three 3D-printed prototypes (stem trap, cylinder traps 1 and 2) and yellow sticky cards, used as a standard (13 cm × 18 cm; Alpha Scents Inc., West Linn, OR, USA). Within each block, 5 m treatment plots were designated. Traps were rotated through each of the four plots during subsequent replications. Traps were hung at breast height and deployed on trees having new flush growth, observable ACP activity, and optimal sunlight during daytime hours.

In Florida, the first set of field experiments were conducted using the stem trap, cylinder trap 1, and yellow sticky cards, deployed at 5–9 day intervals between 1 May 2018 and 26 June 2018. Capture vials were monitored after each experiment, and the number of ACP trapped was documented. Clear plastic food wrapping was folded and cut around sticky cards to contain the gummy substance during transport. The number of ACP trapped during each deployment was counted from the sticky cards and preservation vials for Merritt Island (*n* = 48) and Ocklawaha (*n* = 72). In the 2nd experiment, 4 types of traps, including the cylinder trap 2, were deployed weekly for 5–9 days between 17 July 2018 to 5 December 2018 at Merritt Island (*n* = 64) and Ocklawaha (*n* = 128).

Data analysis was performed using R statistical computing software (R Core Team, 2018, stats v3.5.2). The null hypothesis was that all traps collect the same numbers of ACP. The data generated follow a Poisson distribution. The Poisson means (ACPs captured per trap, per evaluation period) were compared within each site using the Poisson test [29]:

H_0_: λ_trap1_/λ_trap2_ = 1

where λ_trap1_ = the Poisson mean for trap 1 (usually the sticky card)

H_a_: λ_trap1_/λ_trap2_ > 1

### 2.6. Trap Evaluations in Field Cages in Pomona, CA

In California, preliminary trap evaluations were conducted in field cages in Pomona for four-week periods per trial during May (trial 1), August (2), September (3), November 2018 (trial 4), February, and April of 2019 (trials 5 and 6). The ACP-rearing field cages (maintained by the Citrus Research Board, California) were screened enclosures of 13.7 m × 13.7 m × 13.7 m housing 6 small trees (about 5–8 feet tall) of either “Alemow” (*Citrus macropylla* Wester) or “Eureka lemon” [*Citrus limon* (L.) Osbeck]. The field trees inside the cages were pruned periodically to keep the plant size small. Clean ACPs were maintained inside the field cages for research projects. The field cages were equipped with a double-door entry system to prevent ACPs from escaping. This provided an ideal setting for conducting pilot studies using newly designed traps. Each field cage was estimated to contain about 20,000 to 100,000 ACPs (negative for *C*Las). In each trial, we evaluated three types of traps (stem trap, cylinder traps 1 and 2) in the field cages for four weeks. A total of six trials was carried out from April 2018 to May 2019. For each trial, we utilized 2 field cages and deployed 48 traps. Sixteen traps of each type were used and positioned at the height of about three-to-six feet, deployed around the inner perimeter of the cages. Each tree had four traps hanging from the branches inside the field cages. Since the comparison was between the three trap models previously selected in Florida, we did not utilize yellow sticky traps in these experiments. At the end of each trial, the traps were removed from the trees, and the number of ACPs captured was recorded. The mean number of ACPs captured in each trap for the duration of the trial were compared using a one-way ANOVA and Tukey’s HSD (honestly significant difference test; *p* ≤ 0.05) [30].

### 2.7. Trap Evaluations in Citrus Groves in Temecula, CA

We conducted field evaluations of cylinder trap 1 in four citrus orchards in the Temecula area of southern California (Riverside County). After the initial visual observation of ACP activity on the citrus trees, four sites were chosen. Location 1 had grapefruit trees and was situated next to an organic grove. Location 2 had lemon trees; many abandoned citrus trees surrounded the grove. Locations 3 and 4 had tangerines (cultivar “Tango”) and sweet oranges (cultivar “Valencia”), respectively, and well-managed groves surrounded the selected experimental blocks. Six trials were conducted in locations 1 and 2 between March 2019 and February 2020, and three trials were conducted in locations 3 and 4 between September 2019 and February 2020. Each trial was carried out using 16 traps (cylinder trap 1) deployed on trees in border rows and 8 yellow sticky traps used as controls. We did not conduct trap evaluation trials for about six weeks when pesticides were applied in any of the test sites. The ACP capture vials were collected at the end of each trial and brought to the laboratory for documentation. Yellow sticky traps were covered with plastic wrap before transporting and brought to the lab for counting the trapped ACPs. The mean ACP capture from each trap type at each location was compared using the Mann–Whitney rank sum test [31]. Cylinder traps were washed with detergent and water before the next deployment.

### 2.8. By-Catch

Non-target insects or by-catch for the 3D-printed stem traps and cylinder trap 1 were tallied for the California field trials, but not for the field trials conducted in Florida. Major arbitrary categories tallied captured insects; most of the Hemiptera were identified to the genus or species level. We did not document the by-catch for the yellow sticky cards.

## 3. Results

### 3.1. Field Trials in Merritt Island and Ocklawaha, Florida

**Figure 4:**
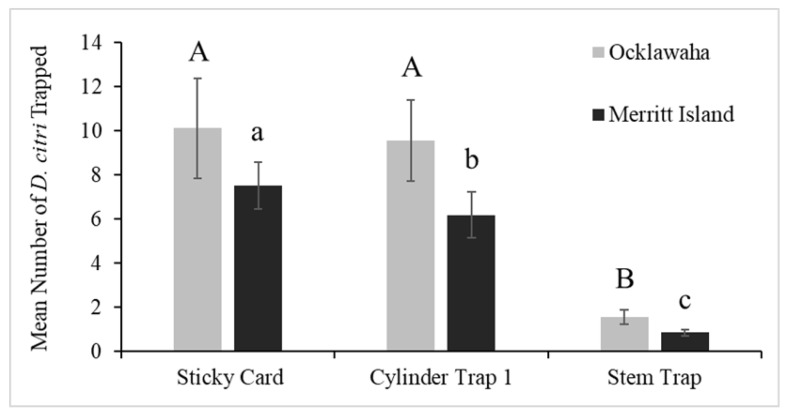
Comparison of ACP capture rates per trap-type in Florida fields using the Poisson test. The three trap-types were deployed in eight blocks per location between 1 May 2018, to 26 June 2018. The number of traps deployed in Merritt Island was 48 and in Ocklawaha was 128. The calculated mean number of psyllids per trap in Ocklawaha were 10.1 for sticky cards, 9.6 for cylinder trap 1 and 1.6 for stem traps. In Merritt Island, the calculated mean numbers were 7.5 for sticky cards, 6.2 for cylinder trap 1 and 0.9 for stem traps. Standard error bars are shown. The use of different letters indicates significant differences in the ACP capture rate within each site (represented by A, B in Ocklawaha and a, b, c in Merritt Island).

Data from the first set of trials conducted between 1 May 2018 and 26 June 2018 suggest that there was no difference between the mean number of ACPs trapped by the sticky cards (10.1) and the mean number of ACPs captured by the cylinder trap 1 (9.6) in Ocklawaha (*p* = 0.300) (Figure 4). The same results were not found in Merritt Island, where the mean number of ACPs trapped by the sticky cards (7.5) was greater than the cylinder trap 1 (6.2) (*p* = 0.016). However, because the 95% confidence intervals calculated for the ratio of these two means are close to one, the means are similar. Conversely, the 95% confidence intervals calculated for the ratio of the mean number of ACPs trapped by the sticky trap (10.1, 7.5) and the stem trap (1.6, 0.9) are not close to one in both Ocklawaha and Merritt Island, and thus the means are very different. Additionally, the mean number of ACPs trapped by cylinder trap 1 (9.6, 6.2) was greater than the mean for the stem trap (1.6, 0.9) in both locations (*p* < 0.001).

**Figure 5:**
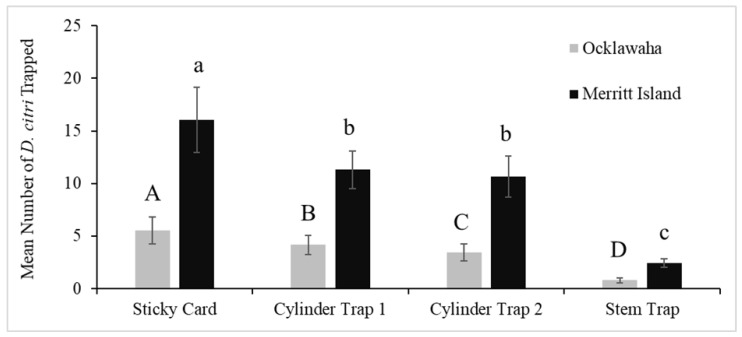
Comparison of ACP capture rates per trap-type in Florida fields using the Poisson test. The three trap-types were deployed in eight blocks per location between 17 July 2018 to 5 December 2018. The number of traps deployed in Merritt Island was 64 and in Ocklawaha was 128. The calculated mean number of psyllids per trap in Ocklawaha were 5.5 for sticky cards, 4.2 for cylinder trap 1, 3.4 for cylinder trap 2, and 0.8 for stem traps. In Merritt Island, the calculated mean numbers were 16.1 for sticky cards, 11.3 for cylinder trap 1, 10.7 for cylinder trap 2, and 2.4 for stem traps. The use of different letters indicates significant differences in the ACP capture rate within each site (represented by A, B, C, D in Ocklawaha and a, b, c in Merritt Island).

The second set of trials included both cylinder traps 1 and 2, evaluated from 17 July 2018 to 5 December 2018. Both designs caught almost as many ACPs as the sticky card in both locations (Figure 5, Supplementary Table S1). Data suggest that there is sufficient evidence to reject the null hypothesis and conclude that the ratio of the two means associated with the sticky card (5.5, 16.1), and cylinder trap 1 (4.2, 11.3) is greater than one for the traps deployed in Ocklawaha (*p* < 0.001) and Merritt Island (*p* < 0.001). Therefore, the mean number of ACPs trapped by the sticky cards was greater than cylinder trap 1. However, because the 95% confidence intervals calculated for the ratio of these two means are close to one, the means are similar. As in previous data, the 95% confidence intervals calculated for the ratios of the mean number of ACPs trapped by the sticky card (5.5, 16.1) and the stem trap (0.8, 2.4) are not close to one. Hence, the means are very different.

Data suggest insufficient evidence to reject the null hypothesis, thus the ratio of the two means associated with cylinder trap 1 (11.3) and cylinder trap 2 (10.7) is equal to one for the traps deployed in Merritt Island (*p* = 0.274). Therefore, the mean number of ACPs trapped by cylinder trap 1 equals cylinder trap 2 in Merritt Island. The same cannot be said for Ocklawaha, where the ratio of the two means associated with cylinder trap 1 (4.2) and cylinder trap 2 (3.4) is not equal to one (*p* = 0.004). However, because the 95% confidence intervals calculated for the ratio of these two means are close to one, the means are similar. Finally, the means for the number of ACPs trapped by cylinder trap 1 (4.2, 11.3) and cylinder trap 2 (3.4, 11.3) were greater than the mean for stem trap (0.8, 2.4) (Figure 5). The Poisson means were compared within each site using the Poisson test.

### 3.2. Trap Evaluations in Field Cages in Pomona, California

**Figure 6:**
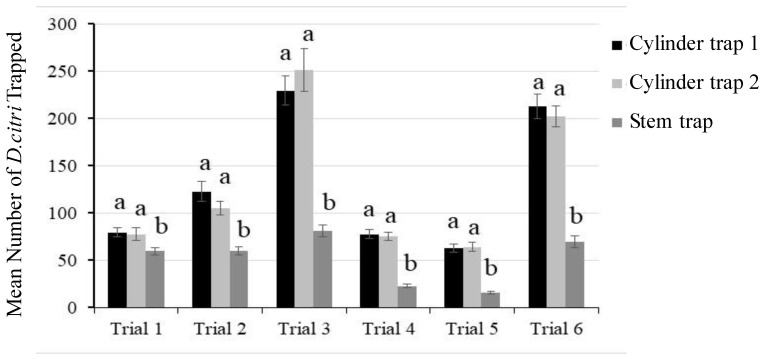
Trap evaluations in field cages in Pomona, California. Six trials were conducted between May 2018 and April 2019, and each trial was conducted for four weeks. A total of 48 traps (16 of each type) were used in 2 field cages per trial. The mean number of ACPs captured per trap, per location for the four-week period is shown. The standard error indicated as bars. The use of different letters indicates significant differences in the ACP capture rate.

ACPs captured in the Pomona field cage trial were counted, and the data were analyzed by comparison using ANOVA and Tukey HSD. The cylinder traps performed better than stem traps in all the trials conducted. Relative values indicate that cylinder traps 1 and 2 performed equally well and were superior to the stem traps (Supplementary Table S2, Figure 6).

### 3.3. Evaluation of Cylinder Trap 1 in Citrus Groves in Temecula, CA

**Figure 7:**
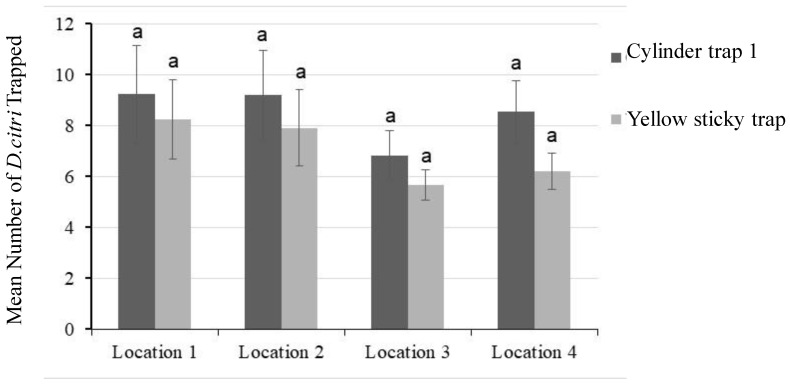
Trap evaluation in citrus groves in Temecula, CA. The mean number of ACPs captured per trap during a six-week evaluation period is shown with standard error bars. Two types of traps were deployed (*n* = 16 for cylinder traps and *n* = 8 for sticky cards). The use of similar letters indicates a lack of significant difference in the ACP capture rates. Field evaluations in Temecula were conducted from March 2019 through February 2020.

For the Temecula field trial evaluations, we used cylinder trap 1 along with yellow sticky traps. The stem traps were determined to be inferior to cylinder traps in previous evaluations, and hence not included in the field trial. Since cylinder traps 1 and 2 had very similar capture rates, only cylinder trap 1 was used. The results indicate that the performance of cylinder trap 1 is equivalent to the yellow sticky trap in all four locations (Figure 7). In locations one and two, the border row adjoining the organic grove or abandoned citrus had more ACPs than traps placed at other locations. However, when the means were calculated, the performance of cylinder trap 1 was at least equivalent to the yellow sticky trap.

### 3.4. By-Catch

In addition to ACPs, the 3D-printed traps (both stem traps and cylinder traps) also captured other arthropods under field conditions. The type of by-catch varied depending on the location, season, weather pattern, pesticide application status, and presence of other plant types adjacent to the citrus grove. The majority of the by-catch consisted of parasitic Hymenoptera, small Diptera, ants, thrips, and small beetles (Table 1). Many of the Hemiptera were identified at least to the genus level. Psyllids other than ACPs collected in the early prototypes of stem traps included *Acizzia uncatoides* (Ferris and Klyver); *Acizzia* spp.; *Bactericera maculipennis* (Crawford); *Calophya schini* Tuthill; *Calophya* spp.; *Ctenarytaina spatulata* Taylor; *Glycaspis brimblecombei* (Moore); and two unknown species (Supplementary Table S3). Aphids were well represented, with 26 species captured in the 2016–2107 trials and 14 species collected in the 2019–2020 trials. In cylinder trap 1 deployed in the Temecula citrus groves (2019–2020), other psyllid species captured included *Acizzia* sp.; *Bactericera cockerelli* (Šulc); *Blastopsylla occidentalis* Taylor; *Cacopsylla* sp.; *G. brimblecombei* (at least 110 individuals); and *Trioza* sp. Among other identified Hemiptera, *Nysius raphanus* Howard (Hemiptera: Lygaeidae) was by far the most frequently collected insect. *Scaphytopius* spp. (Hemiptera: Cicadellidae: Deltocephalinae), *Sophonia orientalis* Matsumura (Hemiptera: Cicadellidae: Evacanthinae), and several Agalline leafhoppers (Hemiptera: Cicadellidae: Megophthalminae) were well represented, especially in the 2019–2020 trials (Supplementary Tables S3 and S4).

### 3.5. Cost of 3D-Printed Traps

In our prototypes, cylinder traps required about 35 g of 3D-printer plastic filament. The cost of producing a cylinder trap, including the price of clear domes, tubes, end caps, collection vials, wire, spray paints, other surface treatments, and the cost of labor, was about USD 16.20 per trap. The stem trap required more 3D-printed components than the cylinder traps (about 200 g). The stem trap could be produced at USD 22.80 per trap.

## 4. Discussion

Citrus HLB caused significant financial damage to citrus industries worldwide. Since there is no known cure for HLB, chemical control of the ACP vector is the primary mode of disease control. The early detection of HLB-associated pathogens is key to mitigating the spread of HLB, especially where the disease is not endemic. In California, the disease is not widespread and hence the industry is still in the disease exclusion mode. Infected trees are removed constantly to decrease the inoculum and prevent further spread of the disease. Enormous resources are utilized for coordinated area-wide monitoring of the ACP vector, and treatment programs for effective disease mitigation follow these.

For a successful integrated pest control program, population monitoring of the insect vector is necessary, since data on pest abundance and spatial distribution are required for effective disease management. Because of the importance of ACP surveys, extensive research has been directed at developing robust monitoring techniques. The presence and abundance of all ACP life stages in citrus trees can be determined by visual inspections of flush shoots [32]. For assessment of adult populations, stem tap sampling is recommended [33]. Other methods employed include sticky traps [27,28,34,35,36], vacuum sampling [37], and sweep nets [38]. Based on comparative studies of the sampling methods reported earlier, yellow or lime-green sticky cards are considered the most effective in monitoring ACP populations, especially at a low density [27,35,36,39].

Yellow sticky traps are used as a standard method for monitoring the insect population in a grove. Integrated pest management programs to suppress ACPs involve the constant monitoring of ACPs, generally accomplished by a combination of yellow sticky cards and tap sampling [40]. Data collected from sticky traps are used for planning pesticide spray regimens to control various insects. However, if an ACP is present in a grove, it would be beneficial to preferentially capture the ACP and test it for the presence of HLB-associated *C*Las. In many disease-survey programs, ACPs are captured manually using aspirators or tap sampling, stored in vials containing alcohol, transported to a laboratory, and used for DNA extractions and testing for the pathogen [25]. While this is an effective method to obtain suitable quality DNA that can be used for molecular testing, manual capture of ACPs is not an efficient process. In addition, the manual method of capture provides pest status in the grove only for the days when ACPs are captured. In an extensive study conducted from samples collected in Pakistan where the high temperatures can reach up to 48 °C for brief periods during the summer, ACPs are generally devoid of the pathogen, as a result of the high temperatures [41]. During extreme weather conditions, the pathogen resides in the host plant’s root system; as normal temperatures resume, the pathogen recolonizes the canopy and can be acquired by the ACP feeding on the plant. For an efficient HLB survey, capturing the ACP vectors during different seasons and evaluating the disease situation is essential. 

**Figure 8:**
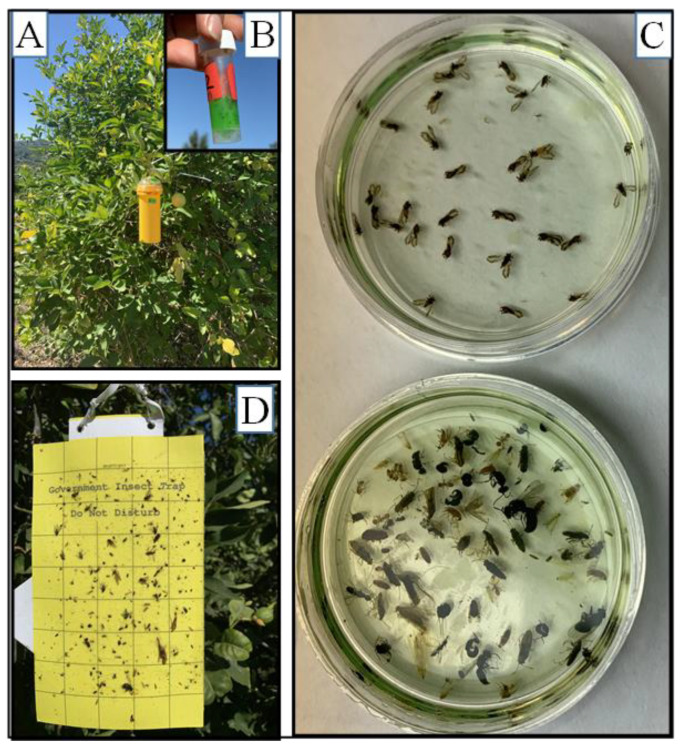
Cylinder trap 1 deployed in a citrus grove in Temecula, CA. Panel (**A**): Trap on a citrus tree. Panel (**B**) (inset) shows the capture tube from the trap with insects. Panel (**C**) shows 28 ACPs (top Petri dish) and by-catch (bottom Petri dish) captured in 1 trap during a 6-week period. Psyllids were separated in the lab. ACPs were clearly recognizable six weeks after capture since they were not degraded. Panel (**D**) shows a yellow sticky trap on an adjacent tree; a magnifying lens was required to identify and count the 20 degraded psyllids on the sticky trap.

Inexpensive, effective, and widely available traps improve the efficiency of ACP surveys. This is increasingly important in areas that are not yet widely affected by the disease, but where disease introduction is heavily monitored. Our results demonstrate that 3D-printed cylinder traps can attract and capture ACPs, and, compared to the yellow sticky card, these traps are efficient in preserving captured ACPs (Figure 8). At some sites, cylinder traps had trapping rates comparable to the yellow sticky cards, making these traps valuable for ACP population monitoring and disease surveys. In pilot studies, we reported that propylene glycol with salt (NaCl) was suitable for ACP preservation and pathogen detection in stored ACPs for up to six weeks [26]. Using a previous model of the stem trap designed and printed by the Division of Plant Industry, similar studies were conducted with the tomato psyllid, *Bactericera cockerelli*; the psyllids were well preserved and suitable for pathogen testing [24].

The 3D-printed trap units can be deployed strategically and in great numbers to cover a large sampling area [42]. Sample vials can be collected from the traps easily and replaced periodically. This reduces the amount of time an inspector has to spend at any given location and maximizes the total area that can be sampled per inspection cycle.

Using 3D printers was essential to the success of this project since it is possible to develop trap prototypes quickly, make required modifications, and evaluate improvements. Initial trap designs were based on data collected by observing ACP behavior in the laboratory setting. Later models incorporated other details, such as the identification of the optimal surface for ACP movement, selection of materials that are non-toxic to the environment, ease of handling, improvement of surface texture so that the ACPs attracted to the trap can move freely on the outer surface of the trap, and the development of a slippery surface to promote movement of captured insects into the collection vial. The development of traps suitable for efficient ACP capture in the field is an iterative process. We described three trap models in this communication. Several modifications were made to the original prototypes to design a trap that is easy to deploy; has apertures large enough for ACPs, but too small to allow larger non-target insects to enter the trap body; is appealing to the target insects; and is durable, using material that the ACP prefers. Preventing other insects from inhabiting the crevices of the trap was incorporated into the later models. The traps can be utilized for other insects and modified depending on the intended use, mainly the type of target insect. The exclusion of specific arthropods may be accomplished by custom modifications, such as the aperture size and other parameters, which may vary depending on the target insect. However, 3D printing performed in a laboratory setting is not be suitable for large-scale production. Once a final design is selected, printing jobs can be outsourced to commercial companies to reduce the price per unit. Since 3D printing is becoming more popular and cheaper, the cost per unit will be significantly lower in the future [43].

Other researchers have utilized 3D-printed traps for various arthropods [22,44,45,46,47]. However, as with any design and manufacturing process, 3D printing does have limitations. Printing times for a standard FDM 3D printer are relatively long, as a single object can take hours to print entirely. Objects can frequently fail to print for many reasons. Print quality and print resolutions can vary significantly among different printer models. Plastic filaments sometimes are printer specific and may be sold in limited colors. We utilized a thermoplastic high-strength polymer, polylactic acid. The filaments selected for this study had a relatively low melting temperature, around 185–190 °C [48].

The stem trap design did not capture ACPs at a rate near to that of the sticky card or cylinder traps. While the stem structures may entice and direct ACPs to walk into the trap, ACPs can just as easily turn around and walk out of the trap. The stem trap is also prone to water intrusion, which reduces the effectiveness of the preservative. The stem trap requires more 3D-printed components than the cylinder traps and requires 12 h of printing and 200 g of plastic filament per trap, increasing the production costs. Due to the complex geometries associated with the stem trap design, it would be unsuitable for mass manufacturing techniques, such as injection molding.

To utilize the cylinder trap effectively for ACP surveys, enough traps should be deployed. Secondly, the traps should be cost-effective. Cylinder trap production can be escalated to reduce trap costs, since effective ACP surveys require the use of a sufficient number of traps per acre. The required numbers may vary according to disease incidence, ACP populations, management practices, etc. In Southern California, where the ACPs have established in citrus groves, 5–16 yellow sticky traps are placed per square mile (5 if a few citrus trees are present and 16 if the density of citrus is high) [25]. Alternate technologies, such as mold injections, can be utilized for producing a large number of traps. All trap components could be mold injected, including the plastic tubes and clear domes. The color of the plastic tubes used in the traps can be matched to a specific spectral signature that is most attractive to ACPs, eliminating the need to hand paint each tube.

Sétamou et al. [20] developed mesh-covered sticky traps to reduce the by-catch of other organisms. Yellow sticky traps often capture many non-target species in addition to small lizards, leaf material, and other debris in the grove. In the 3D-printed traps utilized in the current study, non-target species were monitored and documented (Table 1, Supplementary Tables S3 and S4). The capture of non-target insects was less of an issue with 3D traps than with sticky traps [22].

For oriental fruit flies (*Bactrocera dorsalis*) and melon flies (*B. cucurbitae*), methyl eugenol (4-allyl-1,2-dimethoxybenzene-carboxylate) and cue lure [4-(p-acetoxyphenyl)-2-butanone], respectively, are used as effective kairomone lures in traps [49]. Such effective attractants are not known for ACPs. Other insect attractants that may increase capture rates include semiochemicals [50], lighting [51,52,53,54] auditory devices [55,56,57,58,59]. Despite research to find odorants attractive to ACP adults [60,61], there are still no effective semiochemicals for luring ACP adults in field population studies. Acetic acid has been reported to increase trap catches of both adult males and females, presumably acting as an ACP pheromone [62]. However, other researchers are yet to confirm the field efficacy of acetic acid. The chemosensory organs on the antennae of ACPs are depauperate [63]. Thus, yellow or lime-green sticky cards that serve as visual attractants for ACPs remain the gold standard in the population studies [27,28,40]. Many insects exhibit positive phototaxis and respond to different wavelengths of light [51]. We observed a similar trend with 3D-printed traps placed at the different sides in the field cages in Pomona, CA. Sunlight positively influenced the capture rate of adult ACPs in the traps. Further research with ACPs may lead to the identification of the correct light conditions for increasing ACP capture in 3D-printed traps.

## 5. Conclusions

We developed two basic models of 3D-printed traps that can be used for ACP capture. The cylinder traps were more efficient in ACP capture than the stem traps. The 3D-printed traps are reusable, and the capture of non-target species in the traps is low compared to the sticky traps. Cylinder traps are at least as efficient as sticky traps and can be combined with sticky traps in routine ACP surveys. Since the 3D-printed traps can be deployed on a tree for an extended period, the captured insects represent dynamic sampling over a period of time. One of the major advantages of using a 3D-printed trap is the ability to test the captured ACPs for the presence of the HLB pathogen, CLas. Unlike sticky traps that can only be used for ACP surveys, 3D traps can also be used for HLB surveys since the captured psyllids are suitable for DNA-based testing.

## Figures and Tables

**Table 1:** List of various arthropods collected in the cylinder traps.

Arthropod Groups Identified	Mean Number Captured per Cylinder Trap
Parasitic Hymenoptera	13.324
Diptera	13.169
Formicidae	9.634
***Diaphorina citri***	**9.084**
Hemiptera (except *D. citri*)	6.676
Thysanoptera	5.648
Coleoptera	4.972
Acari	0.958
Araneae	0.535
Lepidoptera	0.479
Neuroptera	0.155
Psocoptera	0.042

Cylinder traps were deployed in the Temecula area (Riverside County, California) from 2019–2020 (*n* = 71). *Diaphorina citri* Kuwayama (ACPs) were identified and counted; other insects were assigned to arbitrary categories, mostly insect orders. Many Hemiptera were identified at a genus or species level (Supplemental Tables S3 and S4). *Diaphorina citri* numbers are included for comparison.

## Data Availability

Open-source files used for printing the cylinder traps will be made available on the Division of Plant Industry website after publication is accepted (https://www.fdacs.gov/Agriculture-Industry/Science/3D-Printing-Lab, accessed on 13 March 2022).

## Supplementary Materials

The following supporting information can be downloaded at: https://www.mdpi.com/article/10.3390/insects13030295/s1, Table S1: Field trials in Florida: a comparison of the different types of traps. Table S2: A comparison of the traps in the Pomona field cage experiment. Table S3: Identified Hemiptera (excluding *Diaphorina citri* Kuwayama) collected in early prototype stem traps deployed in Riverside and Santa Paula, CA, 2016–2017. Table S4: Identified Hemiptera (excluding *Diaphorina citri* Kuwayama) collected in cylinder traps deployed in Temecula, CA, 2019–2020.
