# Supplementary material for: The Development and Evaluation of Insect Traps for the Asian Citrus Psyllid, Diaphorina citri (Hemiptera: Psyllidae), Vector of Citrus Huanglongbing
Source: Insects. 2022 Mar 16;13(3):295. doi: 10.3390/insects13030295 (PMC8954215; doi:10.3390/insects13030295)
Supplement: Supplementary file 1 [file insects-13-00295-s001.zip › insects-1602519-supplementary.pdf]

Table S1. Field trials in Florida: comparison of different types of traps.					
Location	Trap1	Trap2	*P*-value [a]	Lower 95% Confidence	Upper 95% Confidence Interval [b]
Merritt Islanc	Sticky card	Cylinder trap ver 1	<0.001*	1.2898	1.5637#
	Sticky card	Cylinder trap ver 2	<0.001*	1.3669	1.6330#
	Sticky card	Stem trap	<0.001*	5.5966	7.9041
	Cylinder trap ver 1	Cylinder trap ver 2	0.274	0.9548	1.1804
	Cylinder trap ver 1	Stem trap	<0.001*	3.9219	5.5923
	Cylinder trap ver 2	Stem trap	<0.001*	3.6906	5.2728
Ocklawaha	Sticky card	Cylinder trap ver 1	<0.001*	1.1854	1.4891#
	Sticky card	Cylinder trap ver 2	<0.001*	1.4235	1.8123#
	Sticky card	Stem trap	<0.001*	5.6331	8.6287
	Cylinder trap ver 1	Cylinder trap ver 2	0.004*	1.0634	1.3743#
	Cylinder trap ver 1	Stem trap	<0.001*	4.2208	6.5236
	Cylinder trap ver 2	Stem trap	<0.001*	3.4786	5.4166

[a] *P*-values with a [*] sign suggest trap means that are significantly different.

[b] Confidence intervals with a [#] sign suggest similar trap means.

Table S2**.** Comparison of traps in Pomona field cage experiment.

Trial	*F*-Statistic	ANOVA *P*-value	Traps compared	Tukey's HSD *P*-value
1	4.53	0.0161	Cylindrical traps 1 and 2	0.9687
			Cylindrical trap 1 and stem trap	0.0247
			Cylindrical trap 2 and stem trap	0.0441
2	17.07	<0.001	Cylindrical traps 1 and 2	0.2526
			Cylindrical trap 1 and stem trap	<0.001
			Cylindrical trap 2 and stem trap	<0.001
3	32.92	<0.001	Cylindrical traps 1 and 2	0.6111
			Cylindrical trap 1 and stem trap	<0.001
			Cylindrical trap 2 and stem trap	<0.001
4	65.47	<0.001	Cylindrical traps 1 and 2	0.9188
			Cylindrical trap 1 and stem trap	<0.001
			Cylindrical trap 2 and stem trap	<0.001
5	49.64	<0.001	Cylindrical traps 1 and 2	0.9842
			Cylindrical trap 1 and stem trap	<0.001
			Cylindrical trap 2 and stem trap	<0.001
6	59.79	<0.001	Cylindrical traps 1 and 2	0.7508
			Cylindrical trap 1 and stem trap	<0.001
			Cylindrical trap 2 and stem trap	<0.001

The ANOVA and Tukey's HSD *P*-values compare the means computed for the ACP captured in traps tested at Pomona, CA. Each of the six trials had three trap designs (n=16) and were conducted from May 2018 through April 2019. Each trial was conducted for 28 days inside field cages.

Table S3. Identified Hemiptera (excluding *Diaphorina citri* Kuwayama) collected in early prototype Stem traps in Riverside and Santa Paula, 2016–2017. The table is organized in sections based on Suborder and Infraorder to keep taxonomic groups together.

**Superfamily/Family**	**Genus**	**species**	**Author**
**Auchenorrhyncha/Cicadomorpha**			
Membracoidea/Cicadellidae	*Ceratagallia*	sp.	
Membracoidea/Cicadellidae	*Chlorotettix*	sp.	
Membracoidea/Cicadellidae	*Empoasca*	sp.	
Membracoidea/Cicadellidae	*Graminella*	sp.	
Membracoidea/Cicadellidae	*Homalodisca*	*vitripennis*	Germar
Membracoidea/Cicadellidae	*Osbornellus*	*scalaris*	(Van Duzee)
Membracoidea/Cicadellidae	*Scaphytopius*	sp.	
Membracoidea/Cicadellidae	*Sophonia*	*orientalis*	(Matsumura)
**Auchenorrhyncha/Fulgoroidea**			
Fulgoroidea/Cixiidae	*Melanoliarus*	sp.	
**Heteroptera/Cimicomorpha**			
Cimicoidea/Anthocoridae	*Orius*	sp.	
Cimicoidea/Anthocoridae	*Orius*	*tristicolor*	(White)
Miroidea/Miridae	*Lygus*	*elisus*	Van Duzee
Miroidea/Miridae	*Rhinacloa*	*forticornis*	Reuter
Miroidea/Thaumastocoridae	*Thaumastocoris*	*peregrinus*	Carpintero and Dellapé
Miroidea/Tingidae	*Corythucha*	sp.	
**Heteroptera/Pentatomomorpha**			
Coreoidea/Rhopalidae	*Liorhyssus*	*hyalinus*	(Fabricius)
Lygaeoidea/Geocoridae	*Geocoris*	sp.	
Lygaeoidea/Lygaeidae	*Nysius*	*raphanus*	Howard
Lygaeoidea/Lygaeidae	*Nysius*	*tenellus*	Barber
Lygaeoidea/Lygaeidae	*Xyonysius*	sp.	
Lygaeoidea/Ninidae	*Cymoninus*	sp.	
Pentatomoidea/Pentatomidae	*Holcostethus*	*approximatus*	Parshley
**Sternorrhyncha**			
Aphidoidea/Aphididae	*Acyrthosiphon*	*lactucae*	(Passerini)
Aphidoidea/Aphididae	*Aphis*	*coreopsidis*	(Thomas)
Aphidoidea/Aphididae	*Aphis*	*craccivora*	Koch
Aphidoidea/Aphididae	*Aphis*	*eugeniae* (?)	van der Goot
Aphidoidea/Aphididae	*Aphis*	*fabae*	Scopoli
Aphidoidea/Aphididae	*Aphis*	*gossypii*	Glover
Aphidoidea/Aphididae	*Aphis*	*spiraecola*	Patch
Aphidoidea/Aphididae	*Aphis*	sp.	

Aphidoidea/Aphididae	*Brevicoryne*	*brassicae*	(Linnaeus)
Aphidoidea/Aphididae	*Diuraphis*	*noxia*	(Mordvilko)
Aphidoidea/Aphididae	*Dysaphis*	sp.	
Aphidoidea/Aphididae	*Eulachnus*	sp.	
Aphidoidea/Aphididae	*Greenidea*	*ficicola*	Takahashi
Aphidoidea/Aphididae	*Greenidea*	*psidii*	van der Goot
Aphidoidea/Aphididae	*Hyperomyzus*	*carduellinus*	(Theobald)
Aphidoidea/Aphididae	*Lipaphis*	*pseudobrassicae*	(Davis)
Aphidoidea/Aphididae	*Myzus*	*persicae*	(Sulzer)
Aphidoidea/Aphididae	*Nearctaphis*	sp.	
Aphidoidea/Aphididae	*Rhopalosiphum*	*maidis*	(Fitch)
Aphidoidea/Aphididae	*Rhopalosiphum*	*padi*	(Linnaeus)
Aphidoidea/Aphididae	*Rhopalosiphum*	*rufiabdominale*	(Sasaki)
Aphidoidea/Aphididae	*Sarucallis*	*kahawaluokalani*	(Kirkaldy)
Aphidoidea/Aphididae	*Shivaphis*	*celti*	Das
Aphidoidea/Aphididae	*Tetraneura*	sp.	
Psylloidea/Aphalaridae	*Ctenarytaina*	*spatulata*	Taylor
Psylloidea/Aphalaridae	*Glycaspis*	*brimblecombei*	Moore
Psylloidea/Calophyidae	*Calophya*	*schini*	Tuthill
Psylloidea/Calophyidae	*Calophya*	sp.	
Psylloidea/Psyllidae	*Acizzia*	*uncatoides*	(Ferris and Klyver)
Psylloidea/Psyllidae	*Acizzia*	sp.	
Psylloidea/Triozidae	*Bactericera*	*maculipennis*	(Crawford)
Psylloidea	unknown		

Table S4. Identified Hemiptera (excluding *Diaphorina citri* Kuwayama) collected in cylinder traps deployed in Temecula, CA, 2019–2020. The table is organized in sections based on Suborder and Infraorder to keep taxonomic groups together.

**Superfamily/Family**	**Genus**	**species**	**author**
**Auchenorrhyncha/Cicadomorpha**			
Cercopoidea/Clastopteridae	*Clastoptera*	sp.	
Membracoidea/Cicadellidae	*Agalliopsis*	*variabilis*	Oman
Membracoidea/Cicadellidae	*Alconeura*	sp.	
Membracoidea/Cicadellidae	*Baldulus*	sp.	
Membracoidea/Cicadellidae	*Aceratagallia*	*californica*	(Baker)
Membracoidea/Cicadellidae	*Aceratagallia*	*longula*	(Van Duzee)
Membracoidea/Cicadellidae	*Empoasca*	sp.	
Membracoidea/Cicadellidae	*Graminella*	sp.	
Membracoidea/Cicadellidae	*Graphocephala*	sp.	
Membracoidea/Cicadellidae	*Homalodisca*	*vitripennis*	Germar
Membracoidea/Cicadellidae	*Macrosteles*	sp.	
Membracoidea/Cicadellidae	*Osbornellus*	sp.	
Membracoidea/Cicadellidae	*Scaphytopius*	sp.	
Membracoidea/Cicadellidae	*Sophonia*	*orientalis*	(Matsumura)
**Auchenorrhyncha/Fulgomorpha**			
Fulgoroidea/Delphacidae	*Peregrinus*	*maidis*	(Ashmead)
**Heteroptera/Cimicomorpha**			
Miroidea/Miridae	*Rhinacloa*	*forticornis*	Reuter
Miroidea/Tingidae	*Corythucha*	sp.	
**Heteroptera/Pentatomomorpha**			
Lygaeoidea/Geocoridae	*Geocoris*	sp.	
Lygaeoidea/Lygaeidae	*Nysius*	*raphanus*	Howard
**Sternorrhyncha**			
Aphidoidea/Aphididae	*Aphis*	*gossypii*	Glover
Aphidoidea/Aphididae	*Protaphis*	*middletonii*	(Thomas)
Aphidoidea/Aphididae	*Aphis*	*spiraecola*	Patch
Aphidoidea/Aphididae	*Aphis*	sp.	
Aphidoidea/Aphididae	*Brachycaudus*	sp.	
Aphidoidea/Aphididae	*Hyalopterus*	*pruni*	(Geoffroy)
Aphidoidea/Aphididae	*Hysteroneura*	*setariae*	(Thomas)
Aphidoidea/Aphididae	*Lipaphis*	*pseudobrassicae*	(Davis)
Aphidoidea/Aphididae	*Myzus*	*persicae*	(Sulzer)
Aphidoidea/Aphididae	*Rhopalosiphum*	*maidis*	(Fitch)
Aphidoidea/Aphididae	*Sarucallis*	*kahawaluokalani*	(Kirkaldy)
Aphidoidea/Aphididae	*Sipha*	*maydis*	Passerini
Aphidoidea/Aphididae	*Uroleucon*	sp.	
Psylloidea/Aphalaridae	*Blastopsylla*	*occidentalis*	Taylor
Psylloidae/Aphalaridae	*Glycaspis*	*brimblecombei*	Moore
Psylloidea/Psyllidae	*Acizzia*	sp.	

Psylloidea/Psyllidae	*Cacopsylla*	sp.	
Psylloidea/Triozidae	*Bactericera*	*cockerelli*	(Šulc)
Psylloidea/Triozidae	*Trioza*	sp.	
